# Preparing Collared Peccary (*Pecari tajacu* Linnaeus, 1758) for Reintroduction into the Wild: A Screening for Parasites and Hemopathogens of a Captive Population

**DOI:** 10.3390/pathogens13010047

**Published:** 2024-01-03

**Authors:** Júlia Angélica Gonçalves da Silveira, Simone Magela Moreira, Ariane Flávia do Nascimento, Marco Miguel de Oliveira, Hudson Andrade dos Santos, Letícia Gracielle Tôrres de Miranda Estevam, Carine Rodrigues Pereira, Anna Gabriela Guimarães Oliveira, Mirella Lauria D’Elia, Andreina de Carvalho Araujo, Juliana Macedo Magnino Silva

**Affiliations:** 1 Department of Preventive Veterinary Medicine, Veterinary School, Federal University of Minas Gerais, Belo Horizonte 31270-901, MG, Brazil; annagoliveira@gmail.com; 2 Department of Agrarian Sciences, Federal Institute of Education, Science and Technology of Minas Gerais-Campus Bambuí, Bambuí 38900-000, MG, Brazil; simone.moreira@ifmg.edu.br (S.M.M.); ariane.nascimento@ifmg.edu.br (A.F.d.N.); 3 Biological Science, State University of Minas Gerais-Unit Ituiutaba, Ituiutaba 38302-192, MG, Brazil; marco.oliveira@uemg.br; 4 Department of Parasitology, Institute of Biological Science, Federal University of Minas Gerais, Belo Horizonte 31270-901, MG, Brazil; hudson@icb.ufmg.br (H.A.d.S.); andreinacarvalhoa@hotmail.com (A.d.C.A.); 5 Study Group on Leishmaniases, Fiocruz-Research Center Renê Rachou, Belo Horizonte 30190-002, MG, Brazil; lettestevam@hotmail.com; 6 Department of Veterinary Medicine, Federal University of Lavras, Lavras 37203-202, MG, Brazil; 7 Aiuká-Consultancy for Environmental Solutions, Praia Grande 11725-000, SP, Brazil; mirelladelia.vet@gmail.com; 8 Fauna Protection Coordination of State Forest Institute-IEF, Belo Horizonte 38400-186, MG, Brazil; juliana.silva@meioambiente.mg.gov.br

**Keywords:** parasite load, captive breeding, tayassuidae, biodiversity conservation

## Abstract

The reintroduction of captive animals to the wild helps restore endangered species, but it risks pathogen transmission, harming wild populations. Such transmission can impact the genetic diversity and long-term viability of these populations. This study assessed parasite diversity and load in captive *Pecari tajacu*, a species native to the Americas and culturally significant to Brazilian indigenous culture, prior to reintroduction. Samples from 24 peccaries were analyzed for ectoparasites, hemopathogens, and stool parasites with direct and molecular analysis. Findings showed that various parasites were present. Two peccaries (8.3%) were infested by the adult tick *Amblyomma sculptum*. Six (25.0%) tested positive for *Trypanosoma evansi*, four (16.7%) for hemobacteria of the family Anaplasmataceae, twelve (50.0%) for hemotropic *Mycoplasma*, and seven (29.2%) for *Leishmania braziliensis*. Stool samples indicated multiple parasites, with sixteen (66.7%) peccaries infected by Strongylida order parasites, Spiruridae in three (12.5%), and *Ascaris suum* in one (4.2%) animal. Cysts of *Balantidium* sp. were found in twenty (83.3%), *Entamoeba polecki* in five (20.8%), and *Iodamoeba bütschlii* in two (8.3%) peccaries. To our current knowledge, this is the first global report of *Leishmania braziliensis*, *Iodamoeba bütschlii*, and *Entamoeba polecki* in *P. tajacu*, irrespective of the environment, including both captivity and wild conditions. Some of these parasites are common in domestic animals, and others are zoonotic, indicating potential interspecies pathogen transmission.

## 1. Introduction

*Pecari tajacu* are mammals of the order Artiodactyla, family Tayassuidae, commonly known as peccaries. Peccaries are wild, pig-like, medium-sized mammals native to the Americas whose range extends from the southwestern United States to northern Argentina. In Brazil, they can be found in a variety of habitats, including the Amazon Rainforest, Cerrado (*Brazilian savanna*), and Atlantic Forest, in groups of up to 32 individuals, playing a crucial role in the ecosystems in which they reside [1,2].

The main reasons for the collared peccary population decline in Brazil are habitat loss and hunting pressure. Habitat losses are driven primarily by agricultural expansion, logging, and urbanization, reducing the land area available for collared peccaries to live and feed. The construction of roads and highways causes an increase in accidents, further reducing populations [3,4]. In addition, habitat fragmentation isolates populations, which can lead to inbreeding and reduced genetic diversity [5]. Hunting is also a significant threat, particularly in Brazil, where they are targeted for their meat, leather, and use in traditional medicine [6,7].

In Brazil, the peccary is a species considered important for both ecological and cultural reasons. Ecologically, individuals act as seed dispersers, and as they are herbivores, they contribute to maintaining the diversity and plant structure of the ecosystems where they live. They are also preyed on by several species, including jaguars, puma, and anacondas, and are, therefore, relevant components of food chains. Culturally, they are important to indigenous communities that have used the species for food, medicinal, and religious purposes for thousands of years [8]. Previous studies on the collared peccary in populations from different biomes present in Brazil detected endoparasites such as *B. coli*, *Entamoeba*, Strongylida, and *A. suum* in captive collared peccaries from the Amazon region [9,10] and Strongylida and Spirurida in the captive collared peccaries from Caatinga [11]. Regarding hemopathogens, *Mycoplasma suis* has been found in captive peccaries from the Atlantic Forest [12] and *T. evansi* in the Pantanal [13]. However, there is still a knowledge gap about the different transmission cycles with active circulation of parasites involving collared peccaries and other mammalian species. The significance of peccary in the transmission of these parasites has yet to be measured.

Conservation efforts are needed to ensure the survival and recovery of collared peccary populations in Brazil and elsewhere. Captive breeding and reintroduction programs must take a comprehensive and integrated approach to deal with multiple threats to collared peccaries and their habitats. However, before reintroducing captive animals into nature, it is crucial to assess their parasite load to mitigate the risk of their transmission to wild populations. The release into the wild of these captive-raised animals can pose significant risks when they are infected with parasites. Some of the main risks include the following: (i) Spread of disease: reintroduced animals can spread their parasites to wild populations, leading to disease outbreaks and affecting the health and survival of native wildlife; (ii) Impact on biodiversity: parasites can have a significant impact on the health and reproductive success of native wildlife, leading to a decline in population sizes and reducing biodiversity; (iii) Genetic effects: parasites may reduce the genetic diversity and adaptability of reintroduced populations, which may compromise their long-term viability and resilience; (iv) Interactions with native parasites: reintroduced animals can introduce new parasites into nature, which can interact with and potentially displace native parasites, altering the balance of ecosystems [14,15].

The aforementioned statements are broadly applicable to all pathogens, necessitating comprehensive research involving multiple experts. In the context of the present study, our specific objective was to evaluate the diversity and variety of parasites and hemopathogens in captive-collared peccaries slated for reintroduction into the wild. We anticipate that the results will offer additional insights to inform strategies for mitigating risks and ensuring the successful reintroduction of individuals from this species into their natural habitat. In this way, it will contribute to existing knowledge on the ecology and conservation of *P. tajacu*.

## 2. Materials and Methods

All protocols have been reviewed and approved by the Commission for Ethics in the Use of Animals of the Federal University of Minas Gerais, protocol No. 171/2016, and by the “Chico Mendes Institute of Biodiversity” (ICMBio) (SISBIO 49490-1).

The present study is part of a multidisciplinary work called “Collared Peccaries Project”, which aims to evaluate the potential of a group of captive peccaries for reintroduction in an area of the Atlantic Forest close to their breeding site. The reintroduction process includes sanitary assessment, genetic characterization, ethnozoological studies, assessment of animal behavior, and predator avoidance training before reintroduction.

The soft release process will occur with subsequent monitoring of the herd and its activities, interactions with the environment, native groups of the same species, and humans. Environmental education activities will also be developed for the population surrounding the release area.

The project is relevant to the conservation of the species, as peccary populations in the state of Minas Gerais are in decline.

### 2.1. Study Area and Peccaries Management

Aiming to investigate the parasitic diversity in a captive population for the conservation and restoration of biodiversity, this cross-sectional study involved a sample of 24 healthy collared peccary adults of both sexes, weighing 18.5 ± 5.4 kg (Supplementary Table S1). The animals were kept in Engenho D’Água, a property of approximately 60 hectares of Brazilian Atlantic Forest with ample water availability, part of the Andorinhas Environmental Protection Area that borders the Uaimií State Forest in Ouro Preto and the São Bartolomeu district in Minas Gerais in the southeastern region of Brazil.

Engenho D’Água is licensed with the competent environmental agency under nº 002/2003, process 02015.002962/2003-02 as a wild animal release area and as a commercial breeder of *Agouti paca*, *Hydrochaeris hydrochaeris*, *Pecari tajacu*, and *Pecari pecari* (IBAMA No. 3146.4756/201-CTF: 255722). The continuous forest surrounding the farm represents one of the last refuges for wildlife, forming a large ecological corridor that connects the private natural heritage reserve, the Caraça Sanctuary, to the Cachoeira das Andorinhas Environmental Protection Area and the Uaimií State Forest. The surrounding areas have been completely taken over by iron ore mining companies [16].

The Uaimií State Forest has an area of approximately 4398 hectares and is an important remnant of the Atlantic Forest, which, together with the Itacolomi State Park, the Tripuí Ecological Station, the Cachoeira Environmental Protection Area, and the Andorinhas Municipal Natural Park, forms a mosaic of conservation units that together comprise around 25,000 hectares of protected areas.

The place where the animals stay (paddocks) has a total area of 2.5 ha, with trees and grass delimited with metal mesh.

The animals were fed pumpkin, pig feed, fruits, and vegetables. Industrialized pig feed, comprising cereal grains, soybean meal, vitamins, and minerals, was supplied. The food was stored appropriately, thereby minimizing the risk of serving as a potential source of pathogen contamination. The feed was available in covered troughs, protected from rain and bad weather. Fruits and vegetables were distributed in covered areas and shaded in the paddocks. Water was supplied through automatic cup-type drinkers. Leftover food was collected daily and taken to a compost bin. The drinking fountains were cleaned weekly. A fecal parasitological examination was carried out annually, and if necessary, the animals were dewormed.

### 2.2. Biological Sample Collection

**Figure 1:**
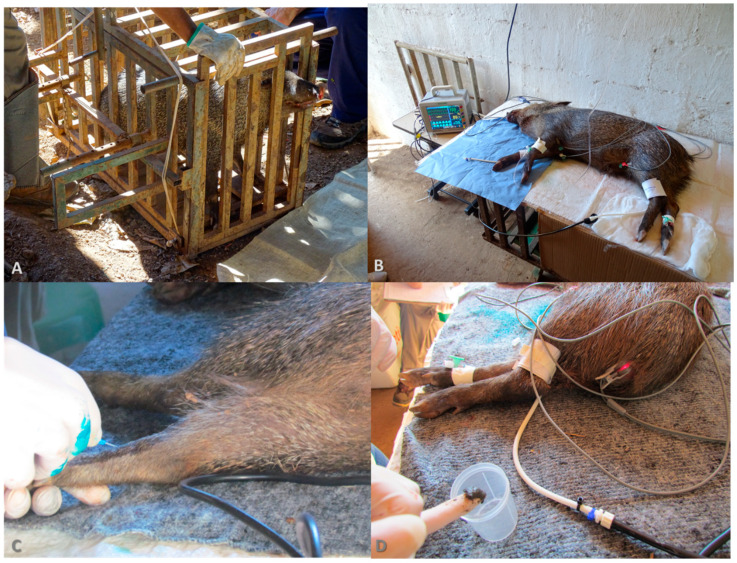
Procedures for sample collection. (**A**) The animal in the containment cage for the application of anesthetics; (**B**) Monitoring the animal after chemical containment; (**C**) Blood collection; (**D**) Feces collection.

For the experimental procedure, the 24 collared peccaries were submitted to an eight-hour fast, with ad libitum access to water. The capture and physical–chemical restraints were carried out as proposed by Silva and collaborators [17]. Capture was carried out by moving the animals to a fixed chute and then to a containment cage. Chemical restraint was conducted using a combination of midazolam (0.41 mg/kg) and butorphanol (0.31 mg/kg) plus detomidine (157 μg/kg) (Figure 1).

All ectoparasites found were removed from the peccaries and stored in 70% ethanol or sent alive to the laboratory. To research ectoparasites, ear swabs were also collected. Feces were collected directly from the rectum of anesthetized animals and placed in containers with parasitological preservatives: one aliquot in 2.5% potassium dichromate for oocyst sporulation and another in MIF (merthiolate-iodine-formaldehyde), at one volume of stool to three volumes of the preservative [18]. Blood was collected via puncture of the cephalic vein, and 5 mL of blood from each animal was collected and packaged without anticoagulants. Blood smears were made with peripheral blood obtained by puncturing the tip of the ear immediately after collection. Skin fragments extracted from the outer surface of the right ear were used for imprints and DNA extraction in the search for *Leishmania* spp. [19].

At the end of the procedures, the animals were transferred to paddocks with an area of 200 m^2^. After allocation, antagonists of the sedative drugs used were administered: atipamezole (350 μg/kg, IM), naloxone (20 μg/kg, IV), and flumazenil (20 μg/kg, IV).

All parasitological analyses were performed at the Federal University of Minas Gerais (UFMG-Brazil).

The ectoparasites were identified using taxonomic keys for ticks [20,21,22].

The collected fecal samples from each animal were divided into two portions: one for flotation examination and the other for sedimentation [23,24,25,26]. The search for helminth eggs, cysts, and protozoan oocysts was performed using an Olympus BX 40 microscope.

- Flotation: A portion will be transferred to Petri dishes containing 2.5% potassium dichromate, properly labeled, where it will undergo maceration and be stored at room temperature for approximately ten days to allow for the sporulation of protozoan oocysts. After this period, 3 mL of this sample will be transferred to a 15 mL Falcon tube containing 12 mL of Sheater’s Solution to separate the feces from potassium dichromate. This material will be centrifuged at 1500 rpm for 15 min. Subsequently, a drop of the supernatant will be placed on a slide and covered with a coverslip for morphometry and identification of oocysts under an Olympus BX 40 Microscope [23].
- Spontaneous sedimentation in water: Another portion of the feces will be strained and transferred to a Hoffman bowl, where it will rest for one to 24 h. Subsequently, a drop of the denser material will be mixed with 10% formalin and placed on a slide, covered with a coverslip for the identification of helminth eggs under an Olympus BX 40 Microscope [24].

For the detection of blood pathogens, analysis of peripheral blood smears and skin imprints following staining, as well as molecular analyses, were performed, as described in the section below.

### 2.3. Integrated Approach of Polymerase Chain Reaction (PCR) and Nucleotide Sequencing for Parasite Detection and Characterization

Genomic DNA was extracted from 300 μL of blood using the Wizard genomic DNA purification kit (Promega^®^, Madison, WI, USA) and from ear tissue samples using the ReliaPrep™ gDNA tissue miniprep system kit (Promega^®^, Madison, WI, USA) per the manufacturer’s instructions. The DNA concentration was quantified using a NanoDrop 1000 Spectrophotometer (Thermo Fisher Scientific, Waltham, MA, USA). All samples were preserved at −20 °C until subsequent molecular analyses.

PCR assays were employed to detect a range of hemopathogens, including various Anaplasmataceae species [27,28,29,30,31,32], hemotropic *Mycoplasma* spp. [33], *Trypanosoma* spp. [34,35,36], *Leishmania* spp. [37,38], and *Babesia*/*Theileria* [39,40] (Table 1). For some hemopathogens, we were able to standardize or adopt nested PCR reactions based on existing literature to enhance diagnostic sensitivity. Reactions employing conventional PCR have been described in the literature, demonstrating both good sensitivity and practical applicability.

PCR amplification followed the protocols standardized by Silveira et al. [41,42]. The initial reaction mixture consisted of 7.5 μL of GoTaq^®^Green Master Mix (Promega, Madison, WI, USA), 0.6 μL of mixed primers (10 mM), and 5.4 μL of nuclease-free water, to which 1.5 μL of total DNA was added to achieve a final volume of 15 μL. The reaction mixtures in the second-round assays were identical, with the first-round PCR products (1.5 μL) serving as templates.

Positive controls, previously confirmed by sequencing, were employed for all target pathogens. These included *Babesia bovis* and *T. vivax* from an experimentally infected calf; *Ehrlichia canis*, *Anaplasma phagocytophilum*, and hemotropic *Mycoplasma* spp. from dogs with confirmed sequences; *T. cruzi*, *T. evansi*, and *Leishmania* sp. (cepas Ba199 (MHOM/BR/1989/Ba199–*L. amazonensis*), BH401 (MCAN/BR/2002/BH401–*L. infantum*), M2904 (MHOM/BR/1975/M2904–*L. braziliensis*), and M4147 (MHOM/BR/1975/M4147–*L. guyanensis*) from experimentally infected mice. DNase and RNase-free Milli-Q water was used as a template control.

Post-PCR, the amplicons were electrophoresed on a 2% agarose gel (40 min; 100 V), stained with GelRed™ (Biotium, Hayward, CA, USA), and visualized under UV light. For *Leishmania* spp. identification, ITS1 amplicons were digested with HaeIII restriction enzyme (New England Biolabs, Ipswich, MA, USA) and analyzed via 5% polyacrylamide gel electrophoresis to establish restriction patterns. These patterns were then compared with WHO (World Health Organization) reference strains [37,38] (Table 2).

Following the second PCR reaction, positive samples were amplified in duplicates to a total volume of 25 μL each, using 50 μL of the amplified product for purification. The samples were purified either via the QIAquick Gel Extraction Kit (QIAGEN, São Paulo, São Paulo, Brazil) following the manufacturer’s instructions or with the Polyethylene Glycol (PEG) method (www.icb.ufmg.br/lbem (accessed on 9 July 2023). Post-purification, each sample underwent another 1% agarose gel electrophoresis, stained with GelRed, to confirm the purity of the samples.

Total genomic material and absorbance ratios (260/280 nm) of the purified product were determined using a spectrophotometer (Nanodrop^®^, Term Scientific, Madison, WI, USA). The purified products were prepared for sequencing following the guidelines provided by Myleus Biotechnology (Belo Horizonte, MG, Brazil) (https://www.myleus.com/ (accessed on 15 February 2021) and ACTGENE-Molecular Analyses Ltda (Alvorada, RS, Brazil) (https://actgene.com.br/ (accessed on 22 May 2022). Sequencing was performed using capillary electrophoresis on an ABI3730 automated sequencer, using BigDye v3.1 polymer and POP7, and the same oligonucleotides used in the PCR assays. The sequencing was carried out once with the forward primer and once with the reverse primer to ensure the reliability of the consensus sequences obtained.

### 2.4. Analysis of Gene Transcript Sequences

Sequences obtained from the sequencer were evaluated for quality, and the contigs were assembled with the aid of the electropherogram quality analysis program developed by Embrapa Genetic Resource and Biotechnology (http://asparagin.cenargen.embrapa.br/phph/ (accessed on 20 February 2021 and 15 June 2021). Subsequently, the sequences were submitted for homology search against sequences deposited in databases using the BLAST program (Basic Local Alignment Search Tool) (http://blast.ncbi.nlm.nih.gov/ (accessed on 20 February 2021 and 15 June 2021). Following the BLAST comparison, the analyzed sequences were classified by gender or species based on the degree of similarity with the data already deposited in GenBank.

Phylogenetic trees were constructed using the partial *16S rRNA* gene nucleotide sequences for *Anaplasma* spp. obtained in this study and those selected from GenBank. Nucleotide sequences were aligned with MUSCLE from the MEGA X package [43]. Following alignment, sequences were evaluated to determine the best DNA models for molecular analysis using the MEGA X package. For *Anaplasma* spp. sequences, each alignment was analyzed using the maximum composite likelihood method (ML) with the Tamura 3-parameter model and the neighbor-joining method (NJ) with the Kimura 2-parameter method. Internal branch confidence was assessed using the bootstrapping method with 1000 replicates. The nucleotide sequences amplified for this parasite in whole blood were deposited in GenBank under the following accession number: PP051538-PP051539.

Given the limited number of animals assessed and parasites found, a descriptive analysis was considered advisable, including percentages and prevalence rates used for data analysis.

## 3. Results

Of the 24 animals sampled, 16 were female, and 8 were male. All the animals presented good body conditions, and none had symptoms compatible with infections, such as diarrhea, dermatitis, and lameness.

**Figure 2:**
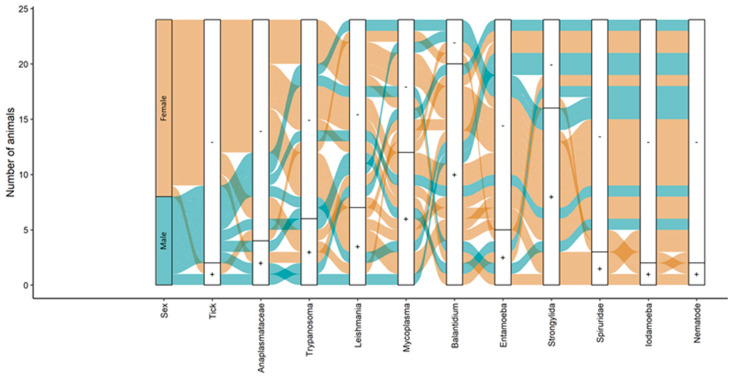
Alluvial diagram showing the distribution of epidemiological characteristics and parasitism in the 24 peccaries analyzed in this study. The + symbol indicates presence, and the - symbol absence. The color blue indicates male, and the color brown indicates female *P. tajacu*.

The results suggest that captive *P. tajacu* may harbor various parasites and hemophatogens (Figure 2).

In the search for ectoparasites, two (8.3%) of the peccaries were found to be infested with the adult tick *Amblyomma sculptum*. Fecal analysis revealed eggs of the order Strongylida in sixteen (66.7%) animals, Spiruridae in three (12.5%), and *Ascaris suum* in one (4.16%) animal. Cysts of *Balantidium* sp. were found in twenty (83.3%) animals, *Entamoeba polecki* in five (20.8%), and *Iodamoeba bütschlii* in two (8.3%) of the peccaries (Supplementary Figure S1).

**Figure 3:**
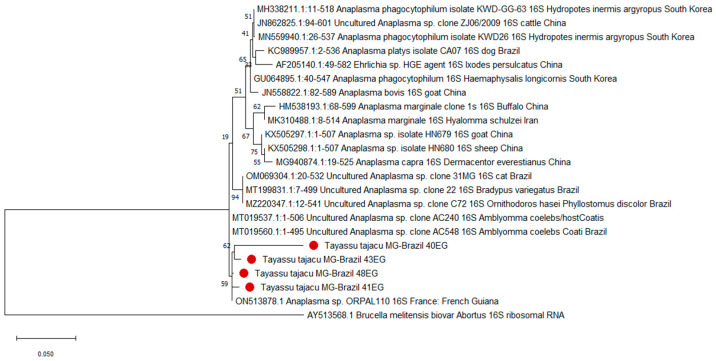
Maximum likelihood tree based on *16S rRNA* genes of Anaplasmataceae isolates. The evolutionary history was inferred using the ML method and Kimura 2-parameter model with gamma distribution and 1000 bootstrap replications. The tree is drawn to scale, with branch lengths measured in the number of substitutions per site. This analysis involved 23 nucleotide sequences. There were a total of 584 positions in the final dataset. Evolutionary analyses were conducted in MEGA X. A sequence from *Brucella melitensis* was utilized to root the phylogenetic trees. Red dots are the sequences from this study. The accession numbers of the publicly available reference sequences are indicated.

In the blood smear and skin imprint analyses, no hemoparasites were observed, suggesting that the evaluated animals had low parasitemia, which provides positive results only in the PCR technique, which is a more sensitive diagnostic method. In the molecular analyses, all animals tested negative for *Trypanosoma vivax* and *T. cruzi*, protozoa of the order Piroplasmida, *Anaplasma marginale*/*A. ovis*, and monocyte hemobacteria of the family Anaplasmataceae (Figure 2). However, it is noteworthy that seven animals (29.2%) were infected with *Leishmania braziliensis* (skin samples), six animals (25.0%) with *T. evansi*, twelve (50.0%) with hemotropic mycoplasmas (Figure 3), and four (16.7%) tested positive for granulocyte/platelet hemobacteria of the family Anaplasmataceae, which are particularly known to cause diseases in animals and humans.

Despite none of the nucleotide sequences observed in this study being 100% identical to known *Anaplasma* sequences, the closest match was at 99.79% (query coverage 94%) with *Anaplasma* sp. from a human, identified in GenBank as ON513878 (Figure 3). The phylogenetic analysis using both maximum composite likelihood (ML) and neighbor-joining (NJ) methods clustered the *Anaplasma* sequences detected in this study into a clade correlated with *Anaplasma* sp., specifically *Candidatus A. sparouinense*, isolated from the human individual (Figure 3).

## 4. Discussion

In this study, we report the diversity and variety of parasites in captive collared peccaries before their reintroduction to the wild. The peccaries had been parasitized by *A. sculptum*, popularly known as the star tick. *A. sculptum* is associated with the presence of preferential hosts such as horses, tapirs, capybaras, and wild pigs [44]. This species is important in public health because it is a vector of *Rickettsia rickettsii*, the agent of Brazilian spotted fever.

Although the animals did not show clinical signs associated with the presence of hemopathogens, in situations of stress or change in management, as in the case of reintroduction, immunosuppression and the appearance of clinical signs may occur. High parasitemia is associated with an increase in the probability of transmission to their vectors during hematophagy. By understanding parasite dynamics in these populations, we can develop effective control measures to minimize the risk of transmission in the wild, protect and restore biodiversity, and improve health outcomes.

*Leishmania braziliensis* is a protozoan parasite that causes American tegumentary leishmaniasis, a neglected tropical disease that affects humans and animals. Its cycle involves phlebotomine sandflies as vectors and wild mammals as reservoirs [45,46]. *Leishmania braziliensis* was not previously reported in *P. tajacu*, either in captivity or in the wild, throughout its whole geographical distribution. In turn, the protozoan *T. evansi* parasitizes a wide range of animals, including domestic and wild mammals. *Trypanosoma evansi* causes a disease known as surra, transmitted in the American continent by dipterans and vampire bats, fomites, or predation [47,48]. In collared peccaries, such infections have been reported in several countries, including Brazil, Colombia, and Venezuela [49,50,51,52]. The presence of these protozoans in captive collared peccaries suggests that despite the peccaries being in captivity, the vectors that transmit them circulate between wild and captive environments.

Species of the Anaplasmataceae family parasitize blood cells and can significantly affect animal health, causing symptoms ranging from fever and anorexia to more serious clinical signs, such as jaundice, weight loss, and even death, in addition to causing great economic damage to production animals, mainly in tropical and subtropical countries [53]. The release into the wild of infected individuals can, therefore, not only compromise the survival of the reintroduced population but also lead to the establishment or resurgence of the disease in the wild. These pathogens usually have complex life cycles involving ticks that collaborate as vectors in the geographic expansion of these pathogens, increasing the risk of exposure of other susceptible wild and domestic animals, and even humans, since some species of Anaplasmataceae are zoonotic.

In the current study, samples of *Anaplasma* sp. isolated from collared peccaries formed a branch with a sample of *Anaplasma* isolated from a human. Despite the discrepancy observed among the samples, we can posit the presence of a potentially new or not fully characterized *Anaplasma* variant. However, further research and genomic analysis are essential to confirm this hypothesis. Importantly, the *Anaplasma* sp. sequence from GenBank originated from a human who had a history of post-traumatic splenectomy, Plasmodium vivax infection in 2019, and a subsequent *Coxiella burnetii* infection in 2021 in the Amazon rainforest of French Guiana. This individual was originally from Maranhão, Brazil, but had been working exclusively in the rainforests of French Guiana for three years [54].

This discovery could have several implications. It could mean that the parasites have a wider host range than previously assumed, potentially affecting conservation efforts and requiring changes to disease management strategies. If this *Anaplasma* sp. is indeed zoonotic, it could also be a risk to human health, particularly for people in close contact with wildlife or those living or working in areas where these animals are present. As such, it underscores the need for continued and more in-depth investigation into Anaplasmataceae infections in both wildlife and human populations, particularly in the context of wildlife reintroduction programs and zoonotic disease surveillance.

On this intriguing issue, it is pertinent to underscore the fact that peccaries frequently serve as hosts to various ectoparasites, including ticks. The identified *A. sculptum* is a notorious species for its role as a vector for a plethora of diseases. The concurrent presence of this tick and a hemopathogen from the Anaplasmataceae family presents a multifaceted network of potential ecological consequences. The phenomenon of co-infection could exacerbate the animal’s health conditions, potentially modifying the trajectory and intensity of the disease, as well as the host’s response, due to complex interactions that might exist between the ticks and pathogens. Beyond the scope of the individual animal, the implications of co-infection extend to population-level dynamics. The simultaneous burden of tick infestation and hemopathogen infection can elevate disease transmission rates among animals and trigger disease outbreaks that ripple through the ecosystem, reshaping mortality patterns and, consequently, transforming community dynamics. Therefore, understanding this co-infection holds paramount importance for wildlife management and disease control strategies.

Hemotropic *Mycoplasma* are a group of bacteria that infect red blood cells and can cause hemolytic anemia in many domestic and wild mammals. The precise routes of its transmission are not yet completely clear, but it appears to be transmitted by blood-sucking arthropods such as ticks, fleas, and lice [55,56]. Hemotropic *Mycoplasma* infection has been reported in mammals from Brazil, including captive white-lipped peccaries (*Tayassu pecari*) and wild boars (*Sus scrofa*) [57,58,59]. In the case of these collared peccaries, exposure to vectors or contaminated materials may explain transmission. Such parasites in captive-bred peccaries may indicate broader biosecurity and management issues in breeding facilities. A comprehensive review of animal care protocols, with a focus on vector control and health monitoring, may be necessary to prevent future outbreaks and ensure animal welfare.

The presence of fecal parasites can be explained by possible errors in management and assistance practices and by the occurrence of parasitism in co-infections due to the overlapping of ecological niches of the vectors and reservoir hosts of these parasites and, in the case of collared peccaries, it may also be related to the environment of captivity, which may facilitate exposure to vectors or other infected animals [60,61].

Strongylida, Spiruridae, and *A. suum* are all types of parasitic nematodes, also known as roundworms, that can infect a variety of hosts, including humans and other animals. These parasites are characterized by their cylindrical, elongated shape and parasitic lifestyle. They infect the gastrointestinal tract and can cause weight loss, anemia, and other gastrointestinal problems. Although they have different specific hosts, they all share a similar life cycle. All of them reproduce through eggs that are released in the feces of their hosts, contaminate the environment, and can be ingested by new hosts. Findings in fecal samples suggest that these parasites may be common in this collared peccary population and that infection may occur through ingestion of contaminated food or water [62]. *Balantidium* sp. is a ciliated protozoan that can cause diarrhea, dysentery, and other gastrointestinal symptoms in animals and humans [63]. *Entamoeba polecki* and *Iodamoeba bütschlii* are also intestinal protozoans that can infect animals and humans, but their clinical significance is not well understood [64]. We could not find previous reports in the literature of *Iodamoeba bütschlii*, *Entamoeba polecki* in *P. tajacu*, regardless of the geographical area.

This parasitic profile can be attributed to the ingestion of contaminated food or water, contact with contaminated feces, and exposure to infected animals or environments [63]. Overcrowding of animals in captive breeding programs can also contribute to the spread of parasites, especially when associated with poor hygiene practices and inadequate waste management [60]. Contaminated water and soil are potential sources of enteroparasites for captive animals. Studies of fecal samples from primates in zoos in Brazil were positive for several enteroparasites, including *Balantidium* sp., whose presence was associated with poor water and soil quality in the breeding environment [65,66].

Feeding practices can also increase the risk of intestinal parasite infection. In research with captive monkeys, several gastrointestinal parasites, including Strongylida and *Ascaris lumbricoides*, were more prevalent among captive animals than those in the wild, indicating that husbandry conditions can significantly affect susceptibility [67]. Furthermore, the handling and housing of animals in captivity may play a role in the transmission of pathogens, and the use of shared equipment and the mixing of animals from different origins can also increase the risk of parasitic infections in captive populations [68].

This research emphasizes the importance of minimizing parasite transmission risks in reintroduction programs. Reintroduction is a crucial conservation strategy to restore populations and support the conservation of endangered species. However, the success of reintroduction programs depends on several factors, including the health and disease status of the reintroduced animals. The health survey of specimens to be reintroduced into the wild is essential for understanding the pathogens that host those individuals before release. This stage of the project aimed only to scan the parasites and blood pathogens of these animals that are candidates for reintroduction. However, a subsequent pre-release survey must be conducted, encompassing the diagnosis of other pathogens such as viruses, bacteria, and fungi.

However, this information may raise doubts regarding the pre-release management of these animals when they are healthy, and the pathogens found are already circulating in the reintroduction region. A preserved environment promotes a dynamic balance in the pathogen–host environment with a high resilience capacity, forming a buffer system for the occurrence of outbreaks [69,70].

If the animals in the present study are reintroduced, it will be in the Uaimií State Forest and its surroundings, an area of preserved environments, but also areas of anthropization such as mining areas and human habitations, which can influence the aforementioned balance, leading to the emergence of diseases by latent pathogens.

The majority of studies on immune responses to soil-transmitted helminths (STH) are conducted using rodent-specific parasites that have been acclimated to the laboratory environment. Following a single high-dose infection, the memory T cells generated persist in the mucosa long after the expulsion of the parasite, providing a basis for mediating protective immunity upon rechallenge [71]. Nevertheless, little is known about the way in which infections are acquired naturally. Therefore, it is not known whether if we treat the animals before infection and eliminate parasitism, the animal may lose its memory immunity, and, in contact with it in the wild, it may become ill (the time of treatment and release must be considered for the loss of response).

Releasing untreated animals poses a risk of environmental contamination, particularly through the release of contaminating forms such as eggs and cysts found in the present study. In this scenario, prior treatment is preferable, as it mitigates the potential for animals to contribute to environmental contamination. We believe that even if the parasites are endemic in the region, treating infected animals is a more manageable task than conducting a comprehensive survey of the presence of these pathogens in that specific location and timeframe.

In relation to ticks, the infestation rate was low (three ticks in two individuals). Therefore, in the new evaluation before reintroduction, if the scenario continues, mechanical removal of the specimens may be sufficient.

In the case of hemopathogens, they were found to have low parasitemia (as they were only detected in PCR and not in blood smears and skin imprints in the case of Leishmania). Therefore, they represent a low risk of contamination from a new hematophagous vector (the main form of transmission of the studied blood pathogens). However, it is important to consider that during the reintroduction process, there may be immunosuppression, and latent parasitemia may become detectable again. Thus, it is important to monitor these animals after release and, if necessary, intervene and treat those peccaries that develop clinical signs or have active parasitemia.

The results provide important information about the diversity and prevalence of parasites in captive animals, and these findings will help in the development of evidence-based strategies to mitigate the risk of parasite transmission during the reintroduction process.

Methodologies for habitat protection, restoration, and creation of corridors can help address the issue of habitat loss and fragmentation, which, in addition to measures to deal with hunting pressure, strengthening regulations, and increasing enforcement and awareness, contribute to the findings presented here, to the formation of a body of knowledge that acts on the ecology and conservation of animals from the vast Brazilian biodiversity. Of particular interest are interdisciplinary approaches to microbiology research, including collaboration with diverse stakeholders such as indigenous communities, which facilitate the development of more comprehensive and effective strategies to control parasites and promote the conservation of endangered species [72].

However, this study has some limitations that must be considered. One of the main limitations is that, although the sample is significant for the number of individuals to be reintroduced, it may not represent what happens in all captive communities. Furthermore, as it was carried out in a specific region of Brazil, the results may not be applicable to other regions. As data were collected from animals born in captivity, continued research is needed to understand parasite dynamics in wild populations. Furthermore, parasite detection was limited to the specific techniques used in the study, and additional techniques could detect other pathogens. Another limitation is the cross-sectional nature of the study, which did not allow for the assessment of temporal trends. Complementary longitudinal studies could provide more information about parasite fluctuations in collared peccaries.

However, this study has some limitations that must be considered. One of the main limitations is that although the sample is significant for the number of individuals to be reintroduced, it may not represent what happens in all captive communities. Furthermore, as it was carried out in a specific region of Brazil, the results may not be applicable to other regions. As data were collected from animals born in captivity, continued research is needed to understand parasite dynamics in wild populations. Furthermore, parasite detection was limited to the specific techniques used in the study, and additional techniques could detect other parasites. Another limitation is the cross-sectional nature of the study, which did not allow for the assessment of temporal trends. Complementary longitudinal studies could provide more information about parasite fluctuations in collared peccaries.

The primary focus of the current study was on conducting a parasitological survey and investigating vector-borne hemopathogens. However, it is imperative to undertake additional studies targeting the identification of various pathogens, including viruses, fungi, and bacteria, before introducing animals into their natural habitat. This is particularly crucial for species with zoonotic potential or importance for domestic animals.

In Brazil, the normative instruction governing releases for reintroduction purposes is outlined in “IN ICMBIO N° 05, OF 13 May 2021” by the national environmental agency. It stipulates that, at a minimum, the pathogens listed in Annex II must undergo testing.

Despite these limitations, this study provides important baseline data on parasite load and diversity in captive *P. tajacu* populations that can be used to inform future research and control strategies. It offers valuable insights into conservation, as well as the importance of working with indigenous Brazilians. Indigenous communities have a long history of using and protecting these animals and their habitats, and their involvement in conservation efforts is critical to the success of these programs [73]. Future studies can take advantage of these insights to develop more comprehensive and effective strategies to control parasites and promote the conservation of endangered species.

## 5. Conclusions

Ultimately, this study provides important information on parasite load and diversity in captive populations of *P. tajacu*, which may have significant implications for programs aimed at restoring threatened populations. Our findings demonstrate a high parasite biodiversity, including some zoonotic ones, suggesting the potential for transmission of pathogens between animals and humans. It also suggests that measures should be implemented to improve hygiene and waste management at breeding sites. More research is needed to investigate other pathogens and their implications for the health and well-being of collared peccaries. By identifying the types and prevalence of parasites present in captive animals, conservationists and wildlife managers can develop effective parasite management protocols to reduce the risk of transmission to wild populations.

## Figures and Tables

**Table 1:** Sequence of primers used to identify the genera of hemopathogens.

Hemopathogen	Sequence (5′→ 3′)	Primer	Target	Products (pb)	References
*Babesia/Theileria* 1st reaction	CGGGATCCAACCTGGTTGATCCTGC CCGAATTCCTTGTTACGACTTCTC	RIB-19 RIB-20	18S rRNA	1700	[39]
2nd reaction	ACCTCACCAGGTCCAGACAG GTACAAAGGGCAGGGACGTA	BAB-rumF BAB-rumR	18S rRNA	430	[40]
*A. marginale*/*A. ovis* 1st reaction	GGGAGCTCCTATGAATTACAGAGAATTGTTTAC CCGGATCCTTAGCTGAACAGGAATCTTGC	MSP45 MSP43	*msp4*	872	[29]
2nd reaction	CGCCAGCAAACTTTTCCAAA ATATGGGGACACAGGCAAAT	AnapF AnapR	*msp4*	294	[30]
*A. phagocytophilum* 1st reaction	ATGAATTACAGAGAATTGCTTGTAGG TTAATTGAAAGCAAATCTTGCTCCTATG	Msp4AP1F Msp4AP1R	*msp4*	-	[31]
2nd reaction	CTATTGGYGGNGCYAGAGT GTTCATCGAAAATTCCGTGGTA	Msp4AP2F Msp4AP2R	*msp4*	450	[32]
Monocytic Anaplasmataceae 1st reaction	ACGGACAATTGCTTATAGCCTT ACAACTTTTATGGATTAGCTAAAT	NS16SCH1F NS16SCH1R	16S rRNA	1195	[27]
2nd reaction	GGGCACGTAGGTGGACTAG CCTGTTAGGAGGGATACGAC	NS16SCH2F NS16SCH2R	16S rRNA	443	[27]
Granulocytic/platelet Anaplasmataceae 1st reaction	CACATGCAAGTCGAACGGATTATTC TTCCGTTAAGAAGGATCTAATCTCC	GE3a GE10r	16S rRNA	932	[28]
2nd reaction	AACGGATTATTCTTTATAGCTTGCT GGCAGTATTAAAAGCAGCTCCAGG	GE9f GE2	16S rRNA	546	[28]
*Trypanosoma evansi* 1st reaction	GCACAGTATGCAACCAAAAA GTGGTCAACAGGGAGAAAAT	Te1F Te1R	ITS	280	[34]
2nd reaction	CATGTATGTGTTTCTATATG	Te2F	ITS	219	[34]
*Trypanosoma vivax*	GCCATCGCCAAGTACCTCGCGA TTAGAATTCCCAGGAGTTCTTGATGATCCAGTA	Tvi2 DTO156	Catepsin L gene	177	[35]
*Trypanosoma cruzi*	AAATAATGTACGGGKGAGATGCATGA GGTTCGATTGGGGTTGGTGTAATATA-	S35 S36	kDNA	333	[36]
*Leishmania* spp.	GGACGAGATCGAGCGCATGGT TCCTTCGACGCCTCCTGGTTG	*hsp*70F *hsp*70R	*Hsp70*	234 pb	[37]
*Leishmania* spp.	CTGGATCATTTTCCGATG TGATACCACTTATCGCACTT	LITSR L5.8S	ITS 1	300–350	[38]
Hemotropic *Mycoplasma* spp.	ATACGGCCCATATTCCTACG TGCTCCACCACTTGTTCA	HBT-F 16S Fw HBT-R 16S Rv	*16S rRNA*	618	[33]

**Table 2:** Fragment sizes (bp) obtained after digestion of targets with the HaeIII enzyme from *Leishmania* spp. controls.

Species	Targets	
	ITS1	HSP70
*Leishmania infantum*	~190, 70 and 60	90, 80 and 70
*Leishmania amazonensis*	~190 and 140	230
*Leishmania braziliensis*	~150 and 140	140
*Leishmania guyanensis*	~150 and 130	180

## Data Availability

The data presented in this research are available in the manuscript and in the supplementary materials.

## Supplementary Materials

The following supporting information can be downloaded at: https://www.mdpi.com/article/10.3390/pathogens13010047/s1, Table S1: Sex of peccaries, body weight, parasites and hemopathogens, individually identified. Figure S1: (**A**–**E**) Eggs and cysts found in fecal samples from peccary. (**A**) Strongylida egg; (**B**) *Ascaris* sp. eggs; (**C**) Spiruridae egg; (**D**) *Entamoeba polecki* cysts; (**E**) *Balantidium* sp. cysts; (**F**) Pair of *Amblyomma sculptum* attached below the tail of a collared peccary.
