# Supplementary material for: Preparing Collared Peccary (Pecari tajacu Linnaeus, 1758) for Reintroduction into the Wild: A Screening for Parasites and Hemopathogens of a Captive Population
Source: Pathogens. 2024 Jan 3;13(1):47. doi: 10.3390/pathogens13010047 (PMC10819336; doi:10.3390/pathogens13010047)
Supplement: Supplementary file 1 [file pathogens-13-00047-s001.zip › pathogens-2786517-supplementary.pdf]

Table S1. Sex of peccaries, body weight, parasites and hemopathogens, individually identified.

Animal Identification	Sex	Body weight (kg)	Ectoparasite	Hemopathogen	Fecal sample
1	M	21		*Trypanosoma evansi*	Strongylida, *Ascaris suum*
2	M	18		*Leishmania braziliensis, Mycoplasma* sp.	*Balantidium* sp.
3	F	17.3		*T. evansi*	Strongylida
4	M	16.1		*Mycoplasma* sp.	*Balantidium* sp., Strongylida
5	F	20		Not detected	*Balantidium* sp., Strongylida
6	F	11.9		*L. braziliensis, Mycoplasma* sp.	*Balantidium* sp., Strongylida
7	F	22.3		Not detected	*Balantidium* sp., Strongylida
8	F	8.7		*Mycoplasma* sp.	*Balantidium* sp., Strongylida
9	F	20.8		Not detected	*Balantidium* sp., Strongylida
10	F	17.2		*Mycoplasma* sp.	*Balantidium* sp., Strongylida
11	F	10.7		Not detected	*Balantidium* sp., Strongylida, *Entamoeba polecki*
12	M	20.2	*Amblyomma sculptum*	*T. evansi,* Anaplasmataceae granulocyte hemobacteria	*Balantidium* sp.

	F	26.3	*T. evansi,* Anaplasmataceae granulocyte hemobacteria	*Balantidium* sp., Strongylida
14	F	21.3	*L. braziliensis*	*Balantidium* sp.
15	F	24.8	*Mycoplasma* sp., Anaplasmataceae granulocyte hemobacteria	*Balantidium* sp., *E. polecki*
16	M	23.1	*L. braziliensis, Mycoplasma* sp.	*Balantidium* sp.
17	F	13.5	not detected	*Balantidium* sp., *E. polecki, Iodamoeba bütschlii*, Strongylida,Spiruridae,
18	F	15	not detected	Strongylida
19	F	23.2	*Mycoplasma* sp.	*Balantidium* sp., *E. polecki*, *I. bütschlii*
20	F	24	*L. braziliensis,* *T. evansi, Mycoplasma* sp., Anaplasmataceae granulocyte hemobacteria	*Balantidium* sp.
21	M	22.5	*Mycoplasma* sp.	*Balantidium* sp., Strongylida, Spiruridae

22	F	17.8		*L. braziliensis*, *T. evansi*, *Mycoplasma* sp.	*Balantidium* sp., Strongylida
23	M	ND		*L. braziliensis*, *Mycoplasma* sp.	*Balantidium* sp., Strongylida
24	F	ND	*A. sculptum*	*L. braziliensis*, *Mycoplasma* sp	*E. polecki*

M= Male; F= Female.

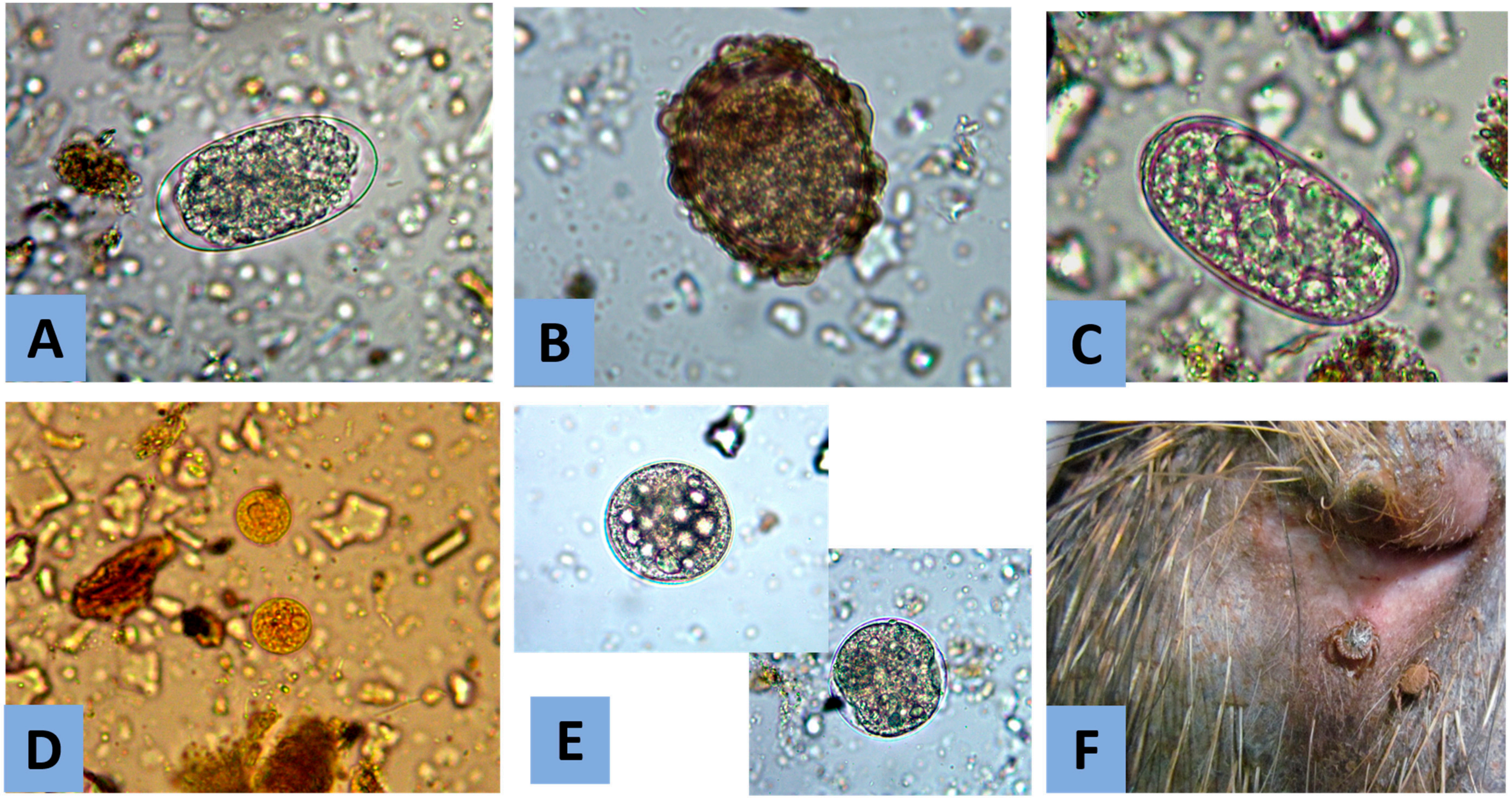

Figure S1. (A–E): Eggs and cysts found in fecal samples from peccary: A) Strongylida egg; B) *Ascaris* sp. eggs; C) Spiruridae egg; D) *Entamoeba polecki* cysts; E) *Balantidium* sp. cysts; F: Pair of *Amblyomma sculptum* attached below the tail of a collared peccary.
